# A Rare Case of Endocarditis and Mycotic Pseudoaneurysm of the Left Ventricle Caused by Escherichia coli Following Transcatheter Aortic Valve Replacement

**DOI:** 10.7759/cureus.27523

**Published:** 2022-07-31

**Authors:** Batel Nissan, Mutaz Karameh, Yonatan Oster, Rabea Asleh

**Affiliations:** 1 Heart Institute, Hadassah Medical Center, Jerusalem, ISR; 2 Clinical Microbiology and Infectious Diseases, Hadassah Medical Center, Jerusalem, ISR

**Keywords:** infective endocarditis, multimodality cardiac imaging, escherichia coli, transcatheter aortic valve replacement, mycotic pseudoaneurysm

## Abstract

Infective endocarditis caused by gram-negative enteral bacteria is very rare. Herein, we report the first case of infective endocarditis complicated by a paravalvular mycotic pseudoaneurysm of the left ventricle caused by *Escherichia coli* post transcatheter aortic valve replacement, highlighting the diagnostic workup, multimodality imaging, and treatment options.

## Introduction

Infective endocarditis (IE) is a life-threatening infection, which can result in cardiac dysfunction, systemic emboli, and mortality. Most of the cases are caused by gram-positive and HACEK (*Haemophilus* species, *Aggregatibacter* species, *Cardiobacterium hominis*, *Eikenella corrodens*, and *Kingella *species) organisms. *Escherichia coli*, a gram-negative enteral bacteria, is a rare cause of endocarditis due to the lack of virulence factors that promote adherence to the endocardial structures. Herein, we report the first case of IE complicated by a paravalvular mycotic pseudoaneurysm of the left ventricle caused by *E. coli* post transcatheter aortic valve replacement (TAVR).

## Case presentation

An 88-year-old man underwent TAVR five years before this current admission due to aortic stenosis followed by transcutaneous pacemaker insertion three years later due to symptomatic bradycardia. In addition, the patient had a history of transitional cell carcinoma (TCC) of the urinary bladder and underwent radical cystectomy with ileal conduit for urinary diversion 20 years prior.

The patient presented with fever and general deterioration for one week. On admission, he was clinically stable with a high-grade fever (38.9°C). Physical examination revealed no focal signs of infection or peripheral signs of IE. Urine and blood cultures were positive for *E. coli*, so a tentative diagnosis of urinary tract infection was suggested and antibiotic treatment with ceftriaxone was started based on sensitivity results. After one week of antibiotic treatment, a significant clinical improvement was observed, serial blood cultures were obtained and were sterile, and he was discharged home. Three days later, the patient was readmitted with similar symptoms, including fever and general deterioration.

Laboratory results on his re-admission showed elevated white blood cell count and C-reactive protein. Kidney function tests were normal; however, metabolic acidosis was noted. Urinalysis was positive for leukocytes, and recurrent growth of *E. coli* in urine and blood cultures was confirmed. Computed tomography (CT) of the head, chest, and abdomen did not show an obvious source of infection and abdominal ultrasound was unremarkable.

**Figure 1:**
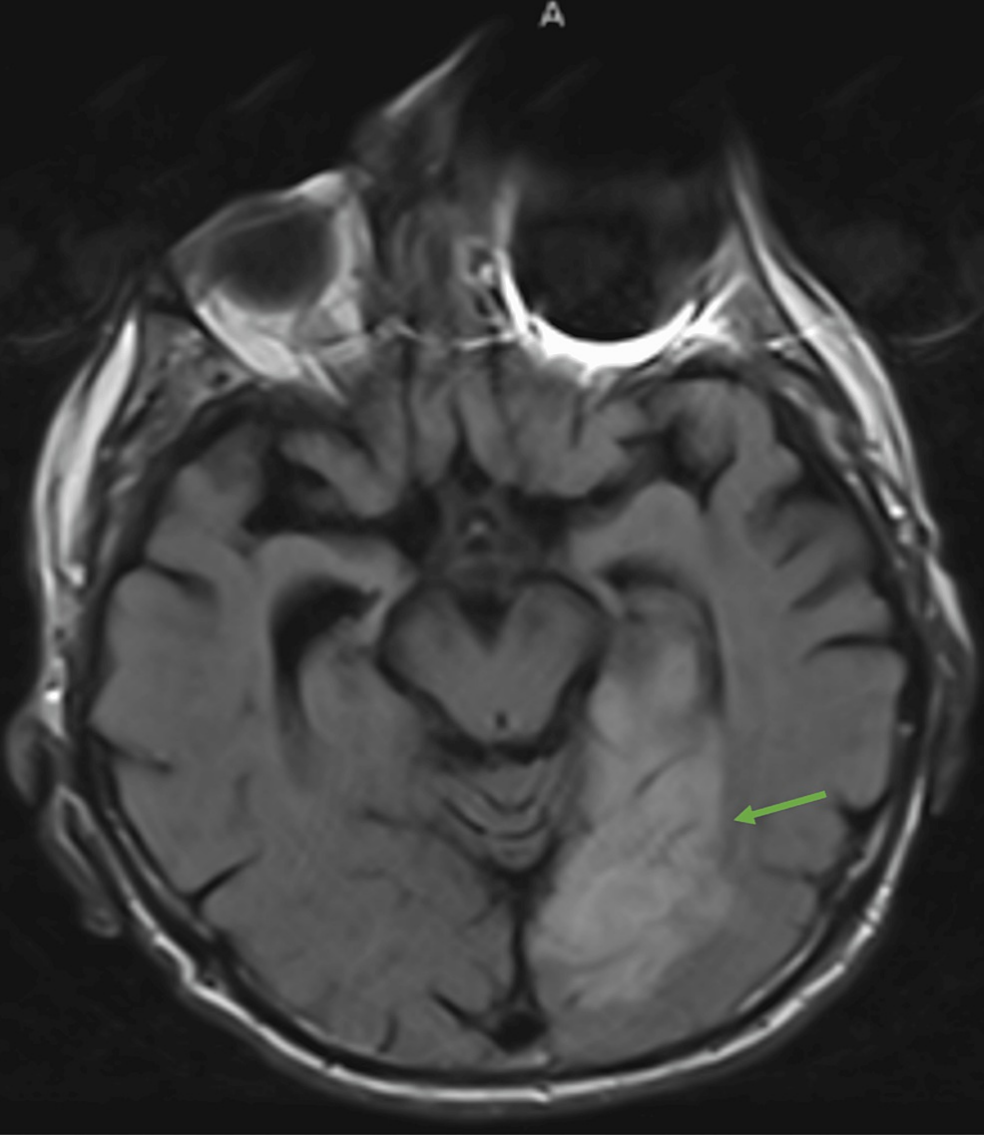
Brain MRI fluid-attenuated inversion recovery (FLAIR) scan (transverse view) showing an acute stroke within the territory of the left posterior cerebral artery (green arrow)

**Figure 2:**
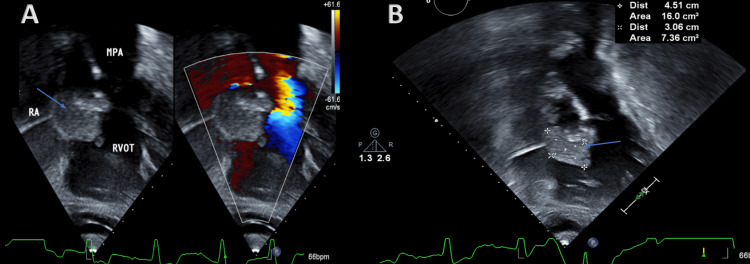
Transthoracic echocardiography (A) Subcostal view showing a hyperechoic mass (blue arrow) protruding from the right atrium (RA) to the right ventricle (RV). Color Doppler imaging was negative for flow within the mass. (B) The mass measurement in two dimensions was 4.51 x 3.06 cm.

During his second hospitalization, he developed transient vision loss in his right eye. Brain magnetic resonance imaging (MRI) revealed acute stroke within the territory of the left posterior cerebral artery with small other infarcts that raised the suspicion of emboli highly suggestive of a cardiac source (Figure 1). Therefore, the patient underwent two-dimensional transthoracic echocardiography (TTE), which revealed an echogenic mass in the right atrium that portended to the right ventricle (Figure 2 and Videos 1, 2).

**Video 1:** Transthoracic echocardiography Four-chambers view showing a mass in the right atrium.

**Video 2:** Transthoracic echocardiography Sub-costal view showing an echogenic mass in the right atrium that portended to the right ventricle.

**Figure 3:**
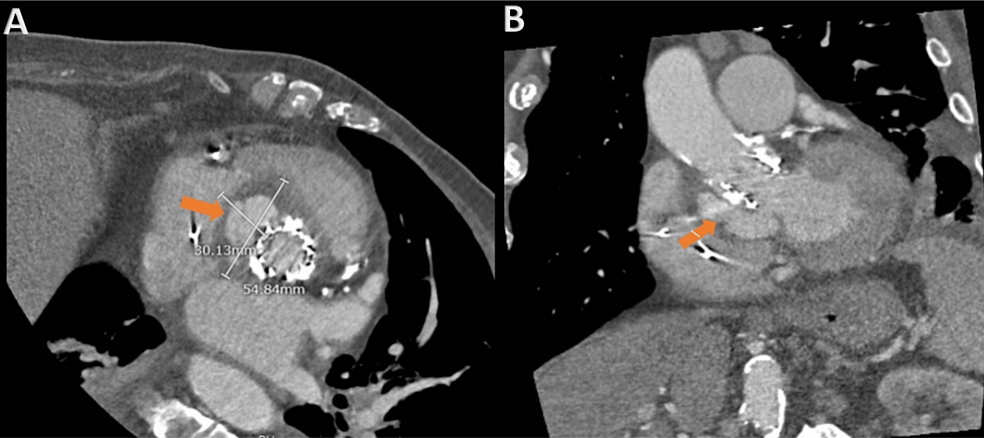
CT scan showing left ventricular pseudoaneurysm (arrows) on axial (A) and coronal (B) views

Given this finding, a cardiac CT was performed demonstrating a pseudoaneurysm posterior to the aortic valve, bulging from the left ventricular outflow tract (LVOT) to the right atrioventricular groove (Figure 3).

**Figure 4:**
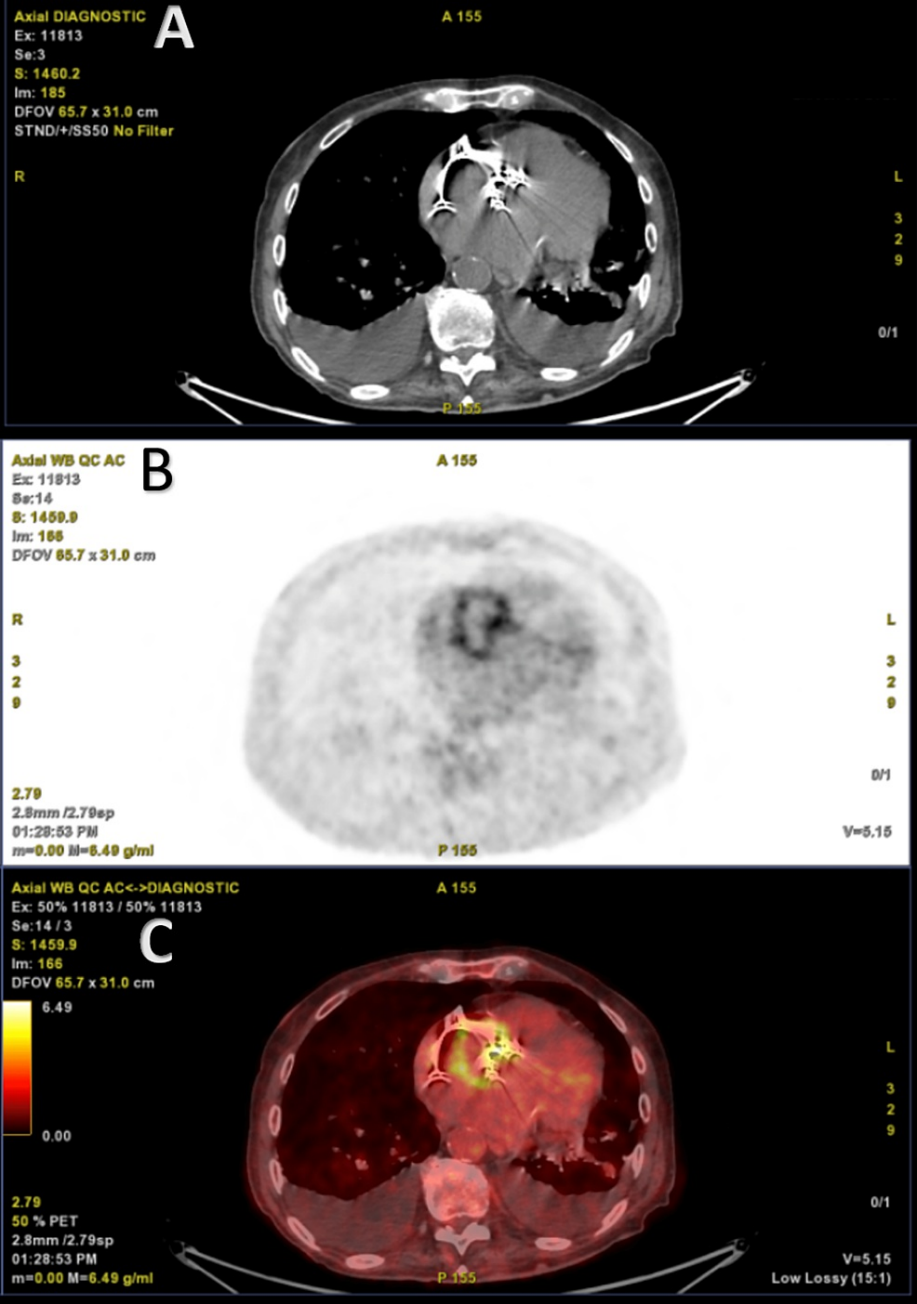
FDG positron emission tomography (PET) scan Transaxial CT scan (A), FDG-PET (B), and fused PET/CT (C) views. ­­There was an increased FDG uptake around the bioprosthetic aortic valve without the involvement of the pacemaker leads. FDG = fluorodeoxyglucose (18F).

Fluorodeoxyglucose (FDG) positron emission tomography (PET) demonstrated high FDG uptake around the aortic valve and within the pseudoaneurysm with no evidence of pacemaker leads' infection (Figure 4).

A multidisciplinary discussion was conducted, including cardiac surgeons, cardiologists, and infectious disease physicians, to discuss the treatment options for the patient with a left ventricle and paravalvular-infected pseudoaneurysm after TAVR. Due to his extremely high surgical risk, the decision was made to continue conservative therapy, including lifelong antibiotic therapy. The patient completed three months of intravenous ceftriaxone followed by oral ciprofloxacin according to bacterial sensitivity testing results.

Follow-up

At a three-month follow-up, the patient had no signs of infection or hemodynamic compromise. However, seven months following the diagnosis of IE, he was readmitted to the hospital due to fever, and blood and urine cultures were positive again for *E. coli* despite oral antibiotic therapy. Unfortunately, resistance testing showed extended-spectrum beta-lactamase (ESBL) profile with resistance to ciprofloxacin; hence, he was treated with intravenous ertapenem. TTE was performed and demonstrated a decrease in mass size. During his hospitalization, the patient developed a *Clostridioides difficile* infection, and despite appropriate antibiotic therapy, his condition deteriorated further and he passed away.

## Discussion

*E. coli* is a gram-negative enteric bacteria and is a rare cause of IE, accounting for less than 0.5% of cases [1]. In 2018, Akuzawa et al. [2] reported 32 cases of endocarditis caused by *E. coli*. Since then, 16 reports of *E. coli* IE on native or prosthetic valves have been added. Common comorbidities related to IE include diabetes mellitus, history of malignancy, excessive alcohol consumption, renal disease, and steroid treatment. *E. coli* IE has been shown to be more common in patients with prosthetic valves; the mitral valve is found to be most affected followed by the aortic valve [2].

The low incidence of *E. coli* IE has been attributed to the lack of virulence factors that promote adherence to the endocardial heart valves and the existence of antibodies against *E. coli* in normal serum [3]. The mortality rate of *E. coli* IE (21%) is higher than IE due to other gram-negative bacteria such as the HACEK group (4%) [1,4]. Generally, urinary tract infections are the common source of *E. coli* IE [2].

The incidence of IE associated with TAVR has been estimated to be 0.8-1.4% [5]. In a recent meta-analysis, no differences in the overall incidence of IE between surgical aortic valve replacement (SAVR) and TAVR were found [6].

The data in the literature regarding the optimal management of *E. coli* IE, whether surgical or conservative, are scarce. In a systematic review of post-TAVR IE, Amat-Santos et al. [7] reported that 60% of patients were managed medically, including those with IE-related complications. The overall in-hospital mortality rate was 34% without significant differences between surgical and conservative approaches. Percutaneous repair of IE-associated complications may be considered in some patients who are not surgical candidates. Ninios et al. [8] reported a case of successful repair of healed endocarditis of the mitral valve using the MitraClip device (Abbott, Abbott Park, IL), despite the presence of large mobile vegetation. Meyer et al. [9] reported a case of successful debriding of the vegetation in a high-risk patient using aspiration-based therapy. Finally, Chan et al. [10] presented an alternative approach to the treatment of flail mitral bioprosthetic valve by valve-in-valve transcatheter mitral valve replacement with an embolic protection device, followed by long-term suppressive oral antibiotic therapy. These reports provide promising therapeutic strategies for percutaneous repair of complications related to IE in high-risk and non-surgical candidates.

## Conclusions

Enteric gram-negative bacteria, particularly *E. coli*, is a rare cause of IE, which can be accompanied by serious complications, particularly after cardiac interventions, such as TAVR. To the best of our knowledge, we report the first case of post-TAVR IE complicated by a mycotic pseudoaneurysm of the left ventricle caused by *E. coli*. Additionally, we highlighted the importance of multimodality imaging in patients with cardiac prosthetic valves or devices to promptly identify and treat IE and its related complications. This case should raise clinicians' awareness of such cases in high-risk patients.
